# Influence of Kinship and MHC Class II Genotype on Visual Traits in Zebrafish Larvae (*Danio rerio*)

**DOI:** 10.1371/journal.pone.0051182

**Published:** 2012-12-10

**Authors:** Cornelia Hinz, Katharina Gebhardt, Alexander K. Hartmann, Lauren Sigman, Gabriele Gerlach

**Affiliations:** 1 Department of Biology and Environmental Sciences, Carl von Ossietzky University of Oldenburg, Oldenburg, Germany; 2 Institute of Physics, Carl von Ossietzky University of Oldenburg, Oldenburg, Germany; 3 Marine Biological Laboratory, Woods Hole, Massachusetts, United States of America; University Zürich, Switzerland

## Abstract

Kin recognition can drive kin selection and the evolution of social behaviour. In zebrafish (*Danio rerio*, Hamilton 1822), kin recognition is based on olfactory and visual imprinting processes. If larvae are exposed to visual and chemical cues of kin at day 5 and 6 post fertilization they will recognize kin throughout life, while exposure to non-kin fails to trigger any recognition. Chemical imprinting signals are transcribed by polymorphic genes of the major histocompatibility complex (MHC) code; however, the underlying mechanism for visual imprinting remains unclear. Here we provide evidence for the existence of family-specific differences in morphometry and pigmentation pattern of six day old zebrafish larvae. While rump, tail and body pigmentation were dependent on relatedness, iris pigmentation and morphometry were also influenced by MHC class II genotype. Our study revealed that the MHC not only influences the chemical signature of individuals, but also their visual appearance.

## Introduction

Kin recognition is a fundamental process for the evolution of cooperative behaviour, driving assortative allocation of resources, mate choice and inbreeding avoidance [1]. Hence, it is not surprising that kin recognition can be found in numerous vertebrate [2], [3], [4] and invertebrate species [5], [6], [7], and even plants [8], [9]. Despite its wide distribution, the sensory cues and neuronal mechanisms of kin recognition are yet not completely understood.

We could show that for later kin recognition zebrafish larvae have to experience an imprinting process that consists of two closely linked steps: a visual imprinting phase on day 5 post fertilization (pf) (unpublished data) followed by the olfactory imprinting phase on day 6 pf [10]. Timing as well as the proper combination of cues are essential because such imprinting only succeeds when both olfactory and visual cues of kin are presented. Larvae exposed to visual or olfactory cues of non-kin on the appropriate days during development failed to imprint (unpublished data). Since these larvae were newly hatched and had never seen or smelled kin, they must innately ‘know’ the visual and olfactory signatures of their kin or be able to compare their own phenotypes to those of unknown individuals and thus be able to avoid imprinting on non-kin.

The highly variable MHC genes are essential for the recognition of extracellular pathogens by the immune system of vertebrates [11]. MHC class II allele similarity is a good indicator for relatedness [12] because it leads to a higher similarity in olfactory cues released via body fluids by related compared to non-related conspecifics. In zebrafish, kin recognition is based on MHC class II genotype similarity; only larvae that share MHC class II alleles can imprint on the olfactory cues of each other (unpublished data). Hence, the predisposition for the olfactory cues of kin can be explained by MHC class II similarity between related individuals.

Despite these findings, the mechanism for the zebrafish’s predisposition to the visual cues of kin remained unclear. We hypothesise that sharing MHC class II alleles not only leads to similarity of olfactory but also to similar visual cues. To prove this hypothesis, we looked for family-and MHC-specific differences in the pigmentation of the body and eyes and in the morphometric appearance parameters of zebrafish larvae.

## Methods

### (a) Study Animals and Rearing Conditions

Adult fish were maintained at 25°C±1°C under a 13/11 light: dark cycle and were fed twice daily with commercial flake food and live brine shrimp (*Artemia salina*). For breeding, each female was kept with one male in a 3 L tank. In the afternoon, egg dishes were placed into the tanks and collected the following morning. All eggs and larvae were maintained in glass dishes in an incubator (SANYO MIR 553) at 25°C±0.5°C. After hatching, which occurred between the 3^rd^ and 4^th^ day pf, larvae were fed with live *Paramecium caudatum*. Altogether, larvae of 5 breeding pairs were tested.

Animal Use and Care Protocols were approved by the Institutional Animal Care and Use Committee of the University of Oldenburg and the government of the state Niedersachsen, Germany (6.12.2007–13.12.2012).

### (b) Analysis of Differences in Morphometric Cues and Pigment Patterns

Using a binocular (Leica MZ 125; 32X magnification) zebrafish larvae were photographed (Canon Eos 5D Mark II) during the imprinting phase and after final establishment of larval pigment pattern (at day 6 pf). Therefore, all larvae were sacrificed using an overdose of MS222 (1 g/100 ml) and immediately positioned on their left side on top of a plexiglas slide in a special larval sized and shaped cavity. All photos were taken within 60 sec after first exposure to MS222 and at the same room temperature to ensure that all larvae experienced the same treatment. After taking the pictures, all larvae were preserved in ethanol for MHC genotyping (see below). The pigment in dead larvae was more intense in colour due to muscle relaxation, but the pattern itself did not differ from that of a live larva when pictures were taken immediately after the death of the animal.

### (c) Morphometry

**Figure 1:**
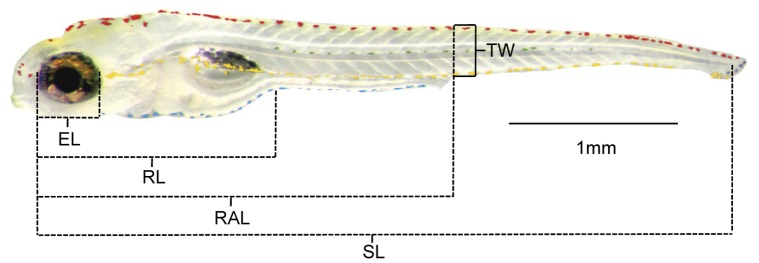
Morphometric measurements and larval body pigmentation with highlighted melanophore stripes*:* red: dorsal stripe; green: lateral stripe; yellow: yolk sack stripe; blue: ventral stripe, SL: standard length (reaching from the nasal periphery of the eye to the caudal periphery of the spine); EL: eye length (reaching from the nasal to the caudal periphery of the eye); RL: rump length (reaching from the nasal periphery of the eye to the border between rump and tail); RAL: rump-anus-length (reaching from the nasal periphery of the eye to the anal opening) and TW: tail width (body width at the anal opening).

Pictures were analysed in ImageJ 1.44p. We measured eye length, rump length, rump-anus length and tail width (Figure 1). All measurements were standardized to standard length (SL). Each measurement was repeated three times and averaged for statistical analysis. Measurements were collected and labelled as follows: k (morphometric measurement) = EL: eye length reaching from the nasal to the caudal periphery of the eye, RAL: rump-anus-length reaching from the nasal periphery of the eye to the anal opening, RL: rump length reaching from the nasal periphery of the eye to the border between rump and tail and TW: tail width (see Figure 1). For further analysis we did pairwise comparisons. For each pair (i, j) of individuals, four averaged length (l) values l_k_(i), l_k_(j) and a weighted difference (D^m^) were calculated via D^m^(i, j) = ∑_k_ α_k_|l_k_(i)−l_k_(j)| with weights α_EL_ = 0.4, α_RAL_ = 0.1, α_RL_ = 0.3, and α_TW_ = 0.2.

### (d) Body and Iris Pigmentation

**Figure 2:**
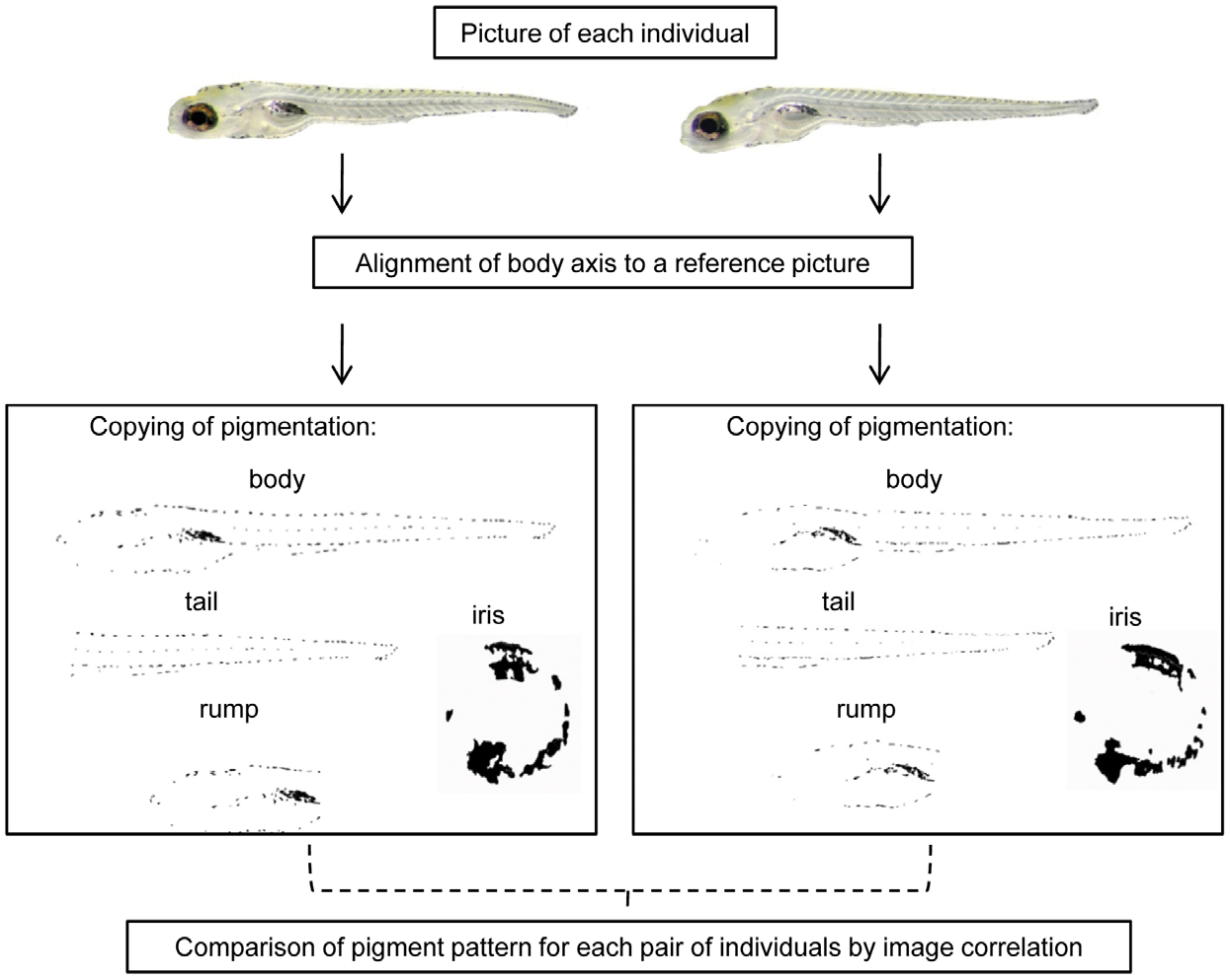
Comparison of iris and body pigmentation between two individuals (Schematic overview).

Using Adobe Photoshop CS2 (Version 9.0.2), pictures of the larvae were aligned so that all were in exactly the same position. The pigmentation of iris and body of each larva was drawn manually in black on a white background using a Wacom Intuos 4 M graphic tablet (Figure 2). The similarity of the distribution pattern of pigmentation was calculated from the drawing pair wise for all individuals (*r*-values) by conducting a 2D correlation in ImageJ 1.44p. The *r*-values spanned from 0–1, with 0 signifying 0% compliance of pixels of two images and 1 signifying 100% compliance. We generated similarity matrices containing the *r*-values for body and iris pigmentation separately for all pairs of individuals. Additionally, we divided the body into three different sections (whole body, rump and tail) and analysed the sections separately in the same way to test whether potential differences in body pigmentation were distributed evenly over the whole body or were limited to specific parts.

### (e) Genotyping of MHC Class II

DNA was extracted from ethanol-preserved fin clips of the parents and from whole larvae. For extraction we used 200 µl of a digestion solution, containing 10% Chelex (Biorad) and 0.07 µg/µl proteinase K. Samples were incubated at 55°C for 1 h, followed by a 10 min heat inactivation step at 95°C. Chelex sedimentation was induced by centrifuging the samples at 15000 rpm for 15 sec and the clear supernatant was transferred into sterile Eppendorf tubes and stored at −18°C until usage.

In zebrafish, two genes (DAA and DAB) represent functional MHC class II loci [13]. We intended to amplify both genes, but null alleles were found in the DAA gene of two of the investigated families. Therefore, we decided to use only DAB. It is important to note that both loci are closely linked. Oligonucleotide primers for amplification Exon 2 (forward primer: TGCATCTACAGCACCAGTGA; reverse primer: CTGCTTTATCACGTACAGCTGA) were designed based on sequences derived from Genbank (accession numbers NM_131476 and AL928944).

The PCR reaction volume was 10 µl, containing 2 µl template DNA, 50 µM of each dNTP, 5 mMMgCl, Q-Solution (Qiagen), 1X PCR buffer (Qiagen), 1 µM of each primer, and 0.25 Units of Hot Star Taq DNA polymerase (Qiagen). PCR conditions were as follows: 15 min at 95°C, 28 cycles (denaturation for 1 min at 94°C, annealing 2 min at 62°C, extension 3 min at 72°C) and final extension at 72°C for 10 min.

To detect allelic differences between MHC class II genes, we used single strand conformation polymorphism (SSCP) analysis, which allows for the separation of DNA fragments that differ by as little as one base pair. 10 µl PCR product was denaturated in 10 µl formamide buffer (95%) for 10 minutes at 96°C und then cooled down immediately to 0°C in ice-water for another 10 minutes. Probes were loaded onto a polyacrylamide gel (9%) containing 1X TBE-buffer. The same buffer was used as running-buffer in the electrophoresis (Biometra Maxi Gel). Electrophoresis was performed at 10°C for 6 h (8 Watt). Gels were stained for 30 min in a staining solution containing 50 ml distilled water and 5 µl GelRed and photographed on a UV transilluminator. By sequencing cut-out DNA bands of the parental PCR products, we verified that similar band patterns on the SSCP gel represent similar alleles.

We genotyped larvae of 5 different mating pairs (n = 20–26 per pair). Two of these pairs shared the same MHC class II while the other pairs differed in all of their MHC alleles. We determined the MHC class II similarity between all photographed individuals according to the number of shared DAB alleles and categorized by 0%, 50% or 100% MHC class II similarity.

### (f) Statistical Analysis

The basic idea of the analysis for similarity of morphometric and pigmentation patterns was to establish relationships via hierarchical clustering. This is a standard approach to represent kin relationships, resulting in ‘dendrogram’ trees with the individuals as leaves of the trees. By clustering different similarity matrices and comparing the resulting dendrograms, we evaluated whether different traits resulted in similar relationships.

First, we changed all similarity (matrix) values (*r)* into distance values (d) via *d* = 1– *r* so they could be used as input for the clustering. For the hierarchical clustering, we applied a standard approach, Wards Algorithm [14], [15], and proceeded as follows. The arrangement of the leaves of a dendrogram is a priori not fully determined, since for each non-leaf node, the order of its two descendants is not determined. Hence, we normalized the arrangements such that the leaf arrangement compared to the original input arrangement (which is ordered by family relationships) resulted in a maximum Goodman-Kruskal’s value [16] 
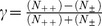
. Here, N_++_ is the number of times where the two individuals of a pair had the same relative order in the arrangement of the input and in the leaves. Correspondingly, N*_+−_* is the number of times the two individuals appeared in a different relative order in the two arrangements. Hence, the dendrograms were arranged such that the original (family) order was as well preserved as possible. Next, we compared all resulting orders of the leaves of the different dendrograms both pair wise and as well as with the input order, by calculating Goodman-Kruskal’s value for each comparison. Finally, to obtain statistical significance, i.e., *p*-values<0.05 [17], we calculated the distribution of values for pair wise comparison of the leaf orders of dendrograms (normalized as above) obtained from 1000 random distance matrices (*D_r_*, “null ensemble”). These *D_r_* matrices were obtained by generating 112 (which is the number of individuals) random points in the square unit and obtaining all Euclidean distances for all pairs of random points. The resulting distribution of values could be well approximated by a Gaussian distribution with mean 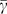 = 0.028 and standard deviation *σ_γ_* = 0.063. Hence, if two orders resulted in a *γ* value larger than 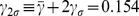, they were likely to be correlated at a probability level of 0.95% (*p*-value<0.05).

## Results

**Figure 3:**
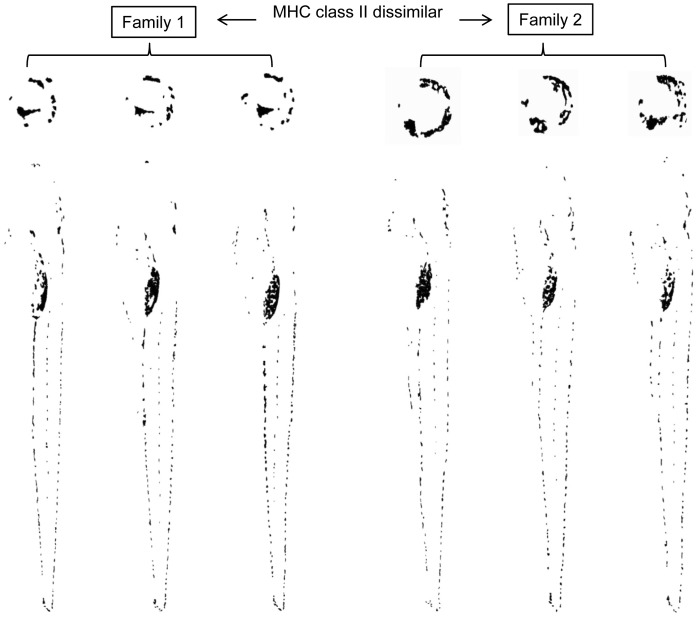
Comparison of iris and body pigmentation between individuals of two MHC class II dissimilar families (shown individuals are MHC class II identical within families and dissimilar between families).

We found that zebrafish larvae who were full siblings were more similar in their morphometric and pigmentation pattern than non-related individuals. An analysis of 12432 pairwise comparisons between 112 individuals revealed that morphometry correlated with family relationship (Goodman- Kruskal`s*γ* (GK): *γ* = 0.298; *p* = 0.000). The same is true for the correlation of the family relationship with different pigmentation patterns, using the r-values from the image correlations, for the iris (GK_iris_: *γ* = 0.489; *p* = 0.000), body (GK_body_: *γ* = 0.329; *p* = 0.000), rump (GK_rump_: *γ* = 0.330; *p* = 0.000) and tail (GK_tail_: *γ* = 0.298; *p* = 0.003), respectively. Furthermore, we found a significant influence of MHC class II similarity on morphometry (GK_morphometry_: *γ* = 0.233; *p* = 0.001) and on pigmentation of iris (GK_iris_: *γ* = 0.458; *p* = 0.000), body (GK_body_: *γ* = 0.323; *p* = 0.000), rump (GK_rump_: *γ* = 0.273; *p* = 0.000) and tail (GK_tail_: *γ* = 0.318; *p* = 0.000) (Figure 3).

**Figure 4:**
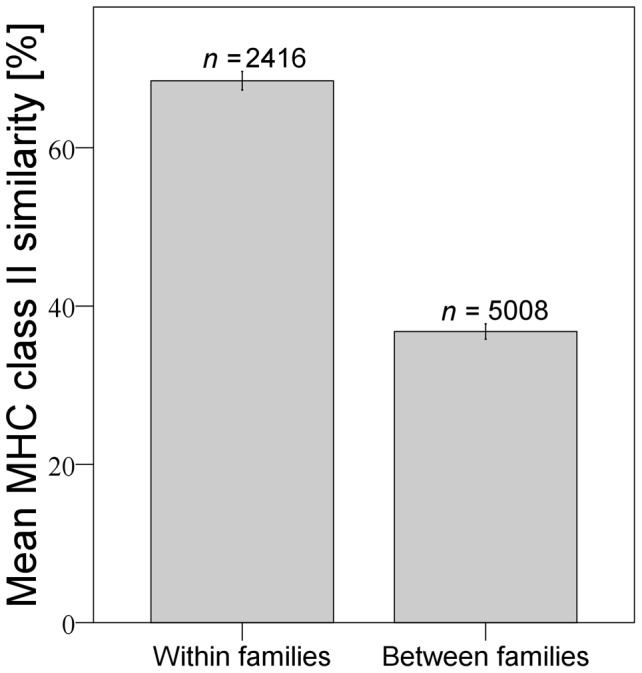
Mean MHC class II similarity within and between families (Error bars: 95% confidence interval).

Because MHC class II similarity was also dependent on relatedness (family effect) (GK: *γ* = 0.761; *p* = 0.000, Figure 4), we conducted a block-wise analysis to investigate which of the two factors is crucial. To investigate the influence of relatedness on the similarity of iris, body, rump and tail pigmentation (represented by *r*-values) and morphometric similarity separately, we analysed only pair wise comparisons between 100% MHC class II identical individuals and conducted independent two-sample t-tests of similarities within and between families. Similarities in iris, body, rump and tail pigmentation and similarities in EL/SL and RL/BL were significantly higher within families than between families (table 1). Since MHC class II similarity between individuals of pair-wise comparisons was 100%, we conclude that other or more genes than MHC class II genes are responsible for these family specific differences.

**Table 1:** Comparison of similarities within and between families between MHC class II identical individuals.

		Mean	*SD*	*N*	*T*	*df*	*p*
**Iris**	Within families	0.45	0.09	1022	9.159	1567.460	0.000*
	Between families	0.41	0.10	741			
**Body**	Within families	0.28	0.07	1022	5.328	1761	0.000*
	Between families	0.26	0.06	741			
**Rump**	Within families	0.39	0.10	1022	3.734	1761	0.000*
	Between families	0.37	0.09	741			
**Tail**	Within families	0.09	0.04	1022	8.701	1761	0.000*
	Between families	0.07	0.03	741			
**EL/SL**	Within families	0.003	0.003	1022	−7.696	1761	0.000*
	Between families	0.005	0.005	741			
**RAL/SL**	Within families	0.015	0.011	1022	1.699	1761	0.089
	Between families	0.015	0.010	741			
**RL/SL**	Within families	0.012	0.010	1022	−6.001	1761	0.000*
	Between families	0.015	0.011	741			
**TW/SL**	Within families	0.004	0.003	1022	−0.455	1569.546	0.649
	Between families	0.004	0.003	741			

(Table shows results of the two-sample t-tests; **SD**: standard deviation; significant results marked with asterisks).

To investigate the influence of MHC similarity independently of relatedness, we conducted a univariate General Linear Model (GLM) using only data from pair wise comparisons within (but not among) families. We tested if *r*-values of pigmentation and differences in morphometric measurements correlate with MHC class II similarity. The analysis showed that the similarities of iris pigmentation, EL/SL and RAL/SL were positively correlated with MHC class II similarity within families (table 1). In contrast, RL/SL was negatively correlated with MHC class II similarity within families. We presume that this negative correlation results from other unknown genetic factors. No significant correlations were found between MHC class II similarity and the other parameters (body, rump and tail pigmentation and TW/SL) within families (table 2). Therefore, we conclude that iris pigmentation, EL/SL and RAL/SL are influenced by MHC class II genotype, while the overall correlation between MHC class II and the body rump and tail pigmentation and RL/SL is probably a by-product of the correlation between family relationship and MHC class II similarity.

**Table 2:** Influence of MHC class II similarity on similarity of pigmentation (r-values) and morphometric measurements (distances) within families.

	MHC class II similarity (%)	Mean	*SD*	*n*	*df*	*F*	*p*
**iris pigmentation**	0	0.39	0.10	130	2	18.895	0.000*
	50	0.44	0.10	1264			
	100	0.45	0.09	1022			
**body pigmentation**	0	0.27	0.07	130	2	1.558	0.211
	50	0.27	0.07	1264			
	100	0.28	0.07	1022			
**rump pigmentation**	0	0.38	0.09	130	2	1.240	0.290
	50	0.38	0.10	1264			
	100	0.39	0.10	1022			
**tail pigmentation**	0	0.08	0.03	130	2	2.436	0.088
	50	0.09	0.04	1264			
	100	0.09	0.04	1022			
**EL/SL**	0	0.0036	0.0022	130	2	4.062	0.017*
	50	0.0038	0.0045	1264			
	100	0.0033	0.0030	1022			
**RAL/SL**	0	0.0193	0.0130	130	2	9.585	0.000*
	50	0.0169	0.0118	1264			
	100	0.0153	0.0111	1022			
**RL/SL**	0	0.0083	0.0066	130	2	9.642	0.000*
	50	0.0112	0.0091	1264			
	100	0.0120	0.0099	1022			
**TW/SL**	0	0.0045	0.0035	130	2	2.166	0.115
	50	0.0041	0.0031	1264			
	100	0.0040	0.0030	1022			

(Table shows results of the univariate General Linear Model (GLM) using only data from pair wise comparisons within (but not among) families; **SD**: standard deviation,**SL**: standard length (reaching from the nasal periphery of the eye to the caudal periphery of the spine); **EL**: eye length (reaching from the nasal to the caudal periphery of the eye); **RL**: rump length (reaching from the nasal periphery of the eye to the border between rump and tail); **RAL**: rump-anus-length (reaching from the nasal periphery of the eye to the anal opening) and **TW**: tail width (body width at the anal opening); significant results marked with asterisks. Note that high r-values represent high similarity between individuals while high distances represent low similarity).

In summary, zebrafish larvae that shared the same MHC class II alleles looked more similar than those that did not share the alleles. Zebrafish larvae showed family-specific differences in all investigated parameters. While body pigmentation was only dependent on genetic relatedness, iris pigmentation and parts of the morphometry (EL/SL and RAL/SL) were additionally influenced by MHC class II genotype.

## Discussion

We showed that zebrafish larvae differ in their visual appearance according to their relatedness. Since zebrafish larvae can visually differentiate between kin and non-kin during the visual imprinting phase (because they do not imprint on the visual cues of non-kin), we assume that 5 days old larvae must be able to recognize those fine differences. Furthermore, we discovered that MHC genes do not only influence the chemical signature but also the visual appearance of zebrafish larvae, offering a possible basis for predisposition for the visual cues of kin.

Because of the relatively large eye size of zebrafish larvae, iris pigmentation is a prominent trait and a good candidate for a key visual imprinting cue. MHC class II genes are expressed in retinal pigment epithelial cells [18], [19] and thus may have some direct influence on iris pigmentation. The process by which MHC genotypes influence pigmentation patterns of the body is unknown, but the concept of MHC genes affecting appearance is not unheard of. For example, it has been shown that heterozygosity at three key MHC loci is associated with facial attractiveness in humans [20]. Furthermore, in three-spined sticklebacks (*Gasterosteus aculeatus*), one MHC class I allele correlates with male redness [21], and in mandrills (*Madrillus sphinx*), specific MHC genotypes correlate with red facial coloration [22].

The requirement of combined visual and chemical stimuli for imprinting might increase the specificity ensuring that zebrafish larvae do not imprint on the wrong cues. Since we showed that zebrafish larvae can olfactory imprint on MHC peptides that also influence social behaviour in mice [23] and sticklebacks [24] the visual cues such as iris pigmentation and morphometric data prevent imprinting on wrong olfactory cues released by other species.

We elucidated an essential step in the imprinting process; however, while our findings help to understand the mechanism for imprinting, some questions remain. Similarity in pigmentation patterns can explain how an individual can differentiate between members of different families, but it cannot explain how naïve zebrafish already ‘know’ the visual appearance of their siblings when they encounter kin for the first time. Two different explanations are conceivable. First, a zebrafish larva could know relatives by recognizing their similarity with its own appearance, or secondly, there could be a genetic predisposition to imprint on conspecifics of a certain appearance. Based on our results, we conclude that it is highly unlikely that a visual self-matching process is involved. A larva might be able to see the caudal part of its body, but definitely not the whole body or its own iris pigmentation. Additionally, it is highly unlikely that zebrafish larvae possess any self-consciousness. Therefore, we regard an innate genetic predisposition for the visual traits of kin as more likely. While olfactory imprinting on MHC-similar kin is easier to understand by assuming that some olfactory receptors might be tuned to MHC signals similar to its own, we assume that the visual template that needs to be activated for visual imprinting on day 5 is encoded in higher brain areas, e.g. in the areas responsible for face recognition [25], [26]. We suggest this pattern recognition mechanism is dependent on MHC class II genotype.

Genetic predispositions for visual traits have been found in other species. When domestic chicks (*Gallus gallus domesticus*) imprint on their mother, they have an innate predisposition for visual stimuli that resemble the shape of a head and neck [27]. However, the predisposition for visual cues of kin that we observed in zebrafish is much finer tuned than what has ever been reported before, and the neuronal mechanism still needs to be examined.

The idea of a genetic predisposition to kin raises an additional important question: If zebrafish larvae already know by genetic predisposition what their siblings look and smell like, why is imprinting necessary at all? One possible explanation is that though MHC class II similarity correlates with relatedness, even full siblings can be MHC class II dissimilar. Since each egg batch contains up to several hundred eggs, there are sure to be some siblings in each batch that are MHC class II dissimilar and therefore would not imprint on each other’s visual and olfactory cues if imprinting was based on a genetic predisposition alone, because this mechanism would be limited to siblings sharing the same MHC class II alleles and would require extended learning of additional cues to develop fail-safe recognition of kin. Furthermore, we presume that during visual imprinting larvae must learn something relevant for the olfactory imprinting process since olfactory imprinting does not occur without visual imprinting occurring first (unpublished data). Larvae might have an innate sensitivity towards the visual traits of MHC class II identical larvae, which are very likely siblings. By imprinting on those individuals, larvae might then learn the visual traits which are independent of MHC class II genotype but family specific, i.e. visual cues that allow the recognition of even MHC class II dissimilar kin. This means that during the imprinting process, the individual learns additional visual and olfactory cues of kin with whom it does not share all MHC class II alleles.

In summary, our results demonstrate the existence of highly family specific traits in 6 day old zebrafish larvae and show that some of those traits depend on MHC class II genotype. Our findings support the idea that MHC genes could be involved in visual imprinting on kin and thus may go far beyond their previously-known functions in the immune system and in olfactory imprinting.
